# West Nile Virus Infection Causes Endocytosis of a Specific Subset of Tight Junction Membrane Proteins

**DOI:** 10.1371/journal.pone.0037886

**Published:** 2012-05-24

**Authors:** Zaikun Xu, Regula Waeckerlin, Matt D. Urbanowski, Guido van Marle, Tom C. Hobman

**Affiliations:** 1 Department of Cell Biology, Li Ka Shing Institute of Virology, University of Alberta, Edmonton, Alberta, Canada; 2 Department of Ecosystems and Public Health, University of Calgary, Alberta, Canada; 3 Department of Microbiology, Immunology and Infectious Diseases, University of Calgary, Alberta, Canada; 4 Department of Medical Microbiology and Immunology, University of Alberta, Edmonton, Alberta, Canada; 5 Li Ka Shing Institute of Virology, University of Alberta, Edmonton, Alberta, Canada; Queen's University, Canada

## Abstract

West Nile virus (WNV) is a blood-borne pathogen that causes systemic infections and serious neurological disease in human and animals. The most common route of infection is mosquito bites and therefore, the virus must cross a number of polarized cell layers to gain access to organ tissue and the central nervous system. Resistance to *trans*-cellular movement of macromolecules between epithelial and endothelial cells is mediated by tight junction complexes. While a number of recent studies have documented that WNV infection negatively impacts the barrier function of tight junctions, the intracellular mechanism by which this occurs is poorly understood. In the present study, we report that endocytosis of a subset of tight junction membrane proteins including claudin-1 and JAM-1 occurs in WNV infected epithelial and endothelial cells. This process, which ultimately results in lysosomal degradation of the proteins, is dependent on the GTPase dynamin and microtubule-based transport. Finally, infection of polarized cells with the related flavivirus, Dengue virus-2, did not result in significant loss of tight junction membrane proteins. These results suggest that neurotropic flaviviruses such as WNV modulate the host cell environment differently than hemorrhagic flaviviruses and thus may have implications for understanding the molecular basis for neuroinvasion.

## Introduction

West Nile virus (WNV) is a blood-borne pathogen that can cause serious systemic and neurological disease in human and animals. In order for this to occur, the virus must cross multiple polarized cell layers after mosquito borne transmission. Resistance to movement of macromolecules and pathogens across epithelia and endothelia is mediated in large part by tight junctions. The latter are apically located protein complexes which are composed of integral membrane proteins including claudins, occludins and junctional adhesion molecules (JAM) that form intracellular interactions with cytoplasmic components such as ZO-1, ZO-2, ZO-3 and the actin cytoskeleton (reviewed in [1]). Homotypic interactions between claudins, occludins and JAMs on apposing cells constitute the main barrier to intercellular passage of macromolecules. Tight junctions are highly dynamic and paracellular permeability can be affected by a variety of physiological and pathological conditions. With respect to the latter, it is thought that the pathogenicity of notable human viruses such as severe acute respiratory coronavirus, influenza and ebola viruses is related to their abilities to negatively impact the barrier function of tight junctions [2], [3], [4]. Interestingly, each of these viruses interferes with tight junctions through different mechanisms.

A number of recent *in vitro* and *in vivo* studies have focused on how WNV crosses polarized cell layers but the collective findings do not agree with respect to the underlying mechanism. For example, one group reported that expression of capsid protein inhibits the barrier function of tight junctions by inducing degradation of claudin proteins in lysosomes [5]. In contrast, Verma et al report that infection of endothelial cells by WNV *per se* does not reduce levels of tight junction components, but rather, matrix metalloproteases that are secreted from infected astrocytes cause breakdown of these structures [6], [7]. Moreover, they indicate that WNV infection actually results in a small but significant increase in claudin-1 levels. Finally, data from another laboratory which conducted pathogenesis studies in mice, support a role for matrix metalloproteinase 9 in WNV-induced disruption of the blood brain barrier through degradation of basement membranes [8]. However, the effects of viral infection on tight junction components were not investigated in this study.

For the first time, we employed a coordinated study to understand the effects of WNV infection on tight junction proteins in both epithelial and endothelial cells. Our findings indicate that WNV infection results in targeted endocytosis of a specific subset of tight junction membrane proteins followed by microtubule-dependent transport to and degradation in lysosomes. However, in contrast to Medigeshi et al [5], we observed that capsid protein expression alone did not result in degradation of tight junction integral membrane proteins.

## Results

### WNV infection results in degradation of a subset of tight junction membrane proteins

**Figure 1:**
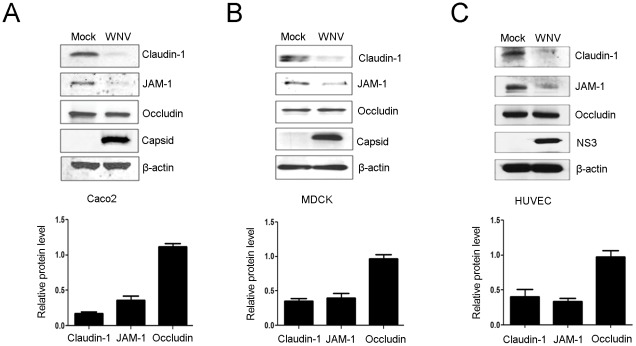
WNV infection results in loss of claudin-1 and JAM-1 proteins in epithelial and endothelial cells. CACO-2 (A), MDCK (B), and HUVEC (C) cells were infected with WNV for 48 hours after which cell lysates were subjected to immunoblot analyses with antibodies to WNV capsid or NS3, claudin-1, JAM-1, occludin and β-actin. The ratios of the relative levels of tight junction proteins (compared to β-actin) from 3 independent experiments were averaged and plotted. Error bars represent standard error of the mean.

Published studies documenting the effects of WNV infection on tight junction complexes are not in agreement. Some of the discrepancies may be due to the fact that one study employed epithelial cells [5] whereas others used endothelial cells [6], [7]. To determine if the published data vary due to cell type specific differences, we analyzed the effects of WNV infection on tight junctions in a number of well characterized epithelial and endothelial cell lines. Data in Figure 1 show that in all cases, the tight junction membrane proteins claudin-1 and JAM-1 are degraded in WNV infected cells. In contrast, levels of occludin protein were unaffected.

**Figure 2:**
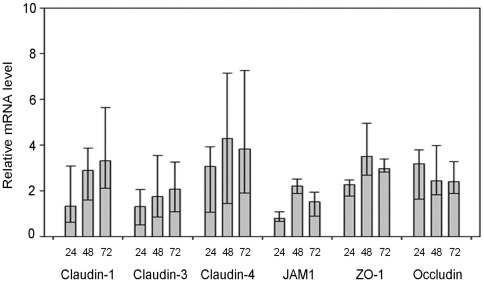
WNV infection leads to increased transcription of multiple tight junction genes. Total RNA extracted from mock and WNV-infected CACO-2 cells at 24, 48 and 72 h post-infection was subjected to quantitative RT-PCR. The levels of tight junction protein encoding mRNAs relative to GAPDH were determined using a comparative cT method. In the mock infected samples, values were normalized to 1.0 but are not shown on the graph. The bars and associated values denominate the mean values, with one-fold standard deviations depicted in the high and low bars.

Lysosomal degradation [5] and matrix metalloproteases [6], [7] have been implicated in WNV-induced turnover of tight junction proteins. However, because a large pool of the WNV capsid protein is targeted to the nuclei of infected cells [9], [10], transcription of claudin-1 and JAM-1 genes could also be affected by WNV replication. Therefore, we used RT-PCR to assess the relative levels of tight junction-specific mRNAs in WNV-infected cells. Data in Figure 2 indicate that WNV infection does not decrease the levels of claudin-1- or JAM-1-specific or other mRNAs that encode tight junction proteins such as claudin-3, claudin-4, ZO-1 and occludin. Instead, levels of tight junction-specific mRNAs were significantly increased as a result of WNV infection. For example, at 24 h post-infection, claudin-1 mRNA levels were >1.8 fold higher than in mock-treated cells and at 72 h post-infection, they were 3.9 times higher (p = 0.039). Claudin-3 and claudin-4 mRNA levels steadily increased during WNV infection and between 48 and 72 h, were as much as 2.2 (p = 0.005) and 4.6 (p = 0.043) fold higher respectively than mock samples. Levels of JAM-1 and ZO-1 mRNAs also increased significantly with peak expression levels observed at 48 h post-infection. Accordingly, we conclude that WNV-induced loss of specific tight junction membrane proteins results exclusively from protein degradation. Moreover, it is likely that this process occurs in all polarized cells regardless of whether they of epithelial or endothelial origin.

### Dynamin and microtubules are required for WNV-induced degradation of claudin-1 and JAM-1

**Figure 3:**
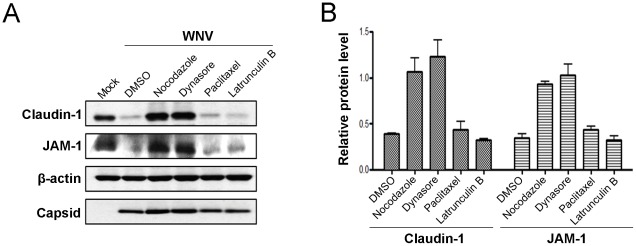
WNV-induced degradation of claudin-1 and JAM-1 requires dynamin and microtubules. A. CACO-2 cells were infected with WNV and 24 hours later were treated with nocodazole (10 µM), Dynasore (10 µM), paclitaxel (1 µM), latrunculin B (10 µM) or DMSO for a further 8 hours. The corresponding cell lysates then subjected to immunoblot analyses. B. Data from three independent experiments were used to determine the normalized levels of claudin-1 and JAM-1 (relative to β-actin).

Having ruled out the possibility that WNV infection affects the transcription and/or degradation of tight junction protein-encoding mRNAs, we next focused on determining how virus infection induces degradation of claudin-1 and JAM-1 proteins. There are a number of ways in which integral membrane proteins of the plasma membrane can be targeted for degradation, the most common of which involves clathrin- or caveolae-dependent endocytosis followed by lysosomal degradation. Moreover, because it has been reported that in response to various physiological and pathological stimuli, tight junction barrier function can be modulated by selective endocytosis of components such as claudins [11], [12], [13], we elected to investigate this pathway first. Internalization of plasma membrane proteins via clathrin-coated vesicles or caveolae requires the action of the GTPase dynamin [14]. As such, if WNV-induced degradation of tight junction membrane proteins involves their removal from the cell surface by canonical endocytic pathways, blocking dynamin function should inhibit the turnover of claudin-1 and JAM-1 in infected cells. Indeed, treatment of cells with the dynamin-specific inhibitor Dynasore [15] completely protected these proteins from degradation during viral infection (Figure 3).

Next, we investigated whether drugs that affect polymerization of actin filaments and microtubules impact the stability of claudin-1 and JAM-1 in WNV-infected cells. Many membrane trafficking events in mammalian cells are dependent upon microtubules and their associated motor proteins (reviewed in [16]), including transport from endosomes to lysosomes [17]. Accordingly, drugs such as nocodazole that inhibit formation of microtubules, should protect claudin-1 and JAM-1 from WNV-induced degradation if the pathway involves transport from endosomes to lysosomes. In contrast to drugs that stabilize microtubules (paclitaxel) or inhibit formation of actin filaments (latrunculin B), treatment of WNV-infected cells with nocodazole completely blocked the degradation of claudin-1 and JAM-1 (Figure 3).

**Figure 4:**
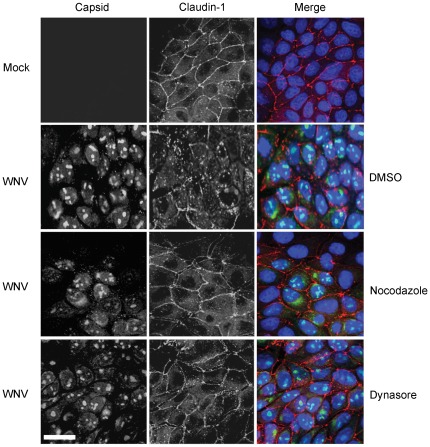
Internalization of claudin-1 is blocked by disrupting microtubules or inhibiting dynamin function. MDCK cells were infected with WNV and 24 hours later were treated with 10 µM nocodazole, 10 µM Dynasore or DMSO for a further 8 hours. Samples were then processed for indirect immunofluorescence using mouse anti-claudin-1 and rabbit anti-WNV capsid. Primary antibodies were detected using donkey anti-mouse Alexa546 and donkey anti-rabbit Alexa488 secondary antibodies. Nuclei were counter stained with DAPI. Images were captured using a Leica TCS SP5 confocal scanning microscope. Size bars are 10 µm.

**Figure 5:**
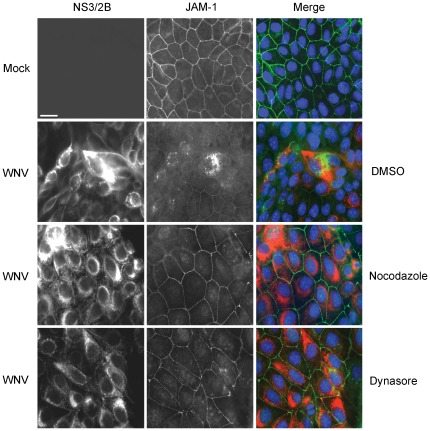
Internalization of JAM-1 is blocked by disrupting microtubules or inhibiting dynamin function. MDCK cells were infected with WNV and 24 hours later were treated with 10 µM nocodazole, 10 µM Dynasore or DMSO for a further 8 hours. Samples were then processed for indirect immunofluorescence using rabbit anti-JAM-1 and mouse anti-WNV NS3/2B. Primary antibodies were detected using donkey anti-mouse Alexa546 and donkey anti-rabbit Alexa488 secondary antibodies. Nuclei were counter stained with DAPI. Images were captured using a Leica TCS SP5 confocal scanning microscope. Size bars are 10 µm.

Together, our data are consistent with a scenario in which WNV infection causes dynamin-dependent endocytosis of claudin-1 and JAM-1 followed by transport along microtubules *en route* to endosomes/lysosomes. However, we cannot rule out the possibility that viral infection causes misrouting of nascent claudin-1 and/or JAM-1 to lysosomes. For example, the nef protein of human immunodeficiency virus downregulates cell surface expression of MHC 1 complexes by stimulating their endocytosis as well as diversion of nascent MHC 1 complexes from the *trans*-Golgi network to the lysomes [18]. Similar to endocytosis, trafficking of proteins along this route is sensitive to nocodazole [19] and also requires dynamin for vesicle scission from the *trans*-Golgi network [20]. Therefore, to differentiate whether WNV infection induces endocytosis of tight junction membrane proteins from the plasma membrane or re-routing of nascent claudin-1 and JAM-1 from the *trans*-Golgi network to the lysosomes, we monitored the localization of these proteins in WNV infected cells that had been treated with Dynasore or nocodazole. If WNV infection causes re-routing of nascent claudin-1 from the *trans*-Golgi network to lysosomes using a mechanism that requires dynamin activity and microtubule-dependent transport, then treatment of infected cells with Dynasore or nocodazole should result in their accumulation in the *trans*-Golgi network and/or associated vesicles in the juxtanuclear region. Based on the data shown in Figures 4 and 5, this does not appear to be the case. When infected cells were treated with these inhibitors, there was no significant build up of claudin-1 or JAM-1 in the juxtanuclear region but rather, we observed that the plasma membrane localization of these proteins was preserved. Therefore, we conclude that the primary mechanism by which WNV induces turnover of tight junction membrane proteins is through dynamin-dependent endocytosis followed by microtubule-dependent transport to lysosomes.

### Dengue virus infection does not affect tight junction membrane proteins

**Figure 6:**
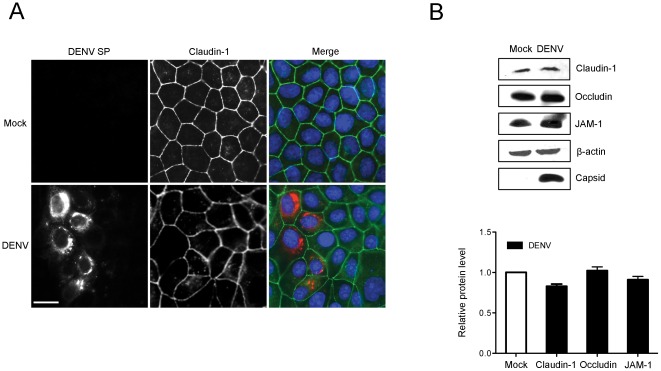
DENV infection does not affect tight junctions. A. MDCK cells were infected with DENV-2 and after 48 hours, samples were processed for indirect immunofluorescence using human anti-DENV serum and mouse anti-claudin-1. The human serum recognizes DENV structural proteins (DENV SP). Primary antibodies were detected using donkey anti-human Texas Red and donkey anti-mouse Alexa488 secondary antibodies. Nuclei were counter stained with DAPI. Images were captured using a Leica TCS SP5 confocal scanning microscope. Size bars are 10 µm. B. CACO2 cells were infected with DENV-2 and after 48 hours, relative levels of claudin-1, JAM-1, and occudin were determined by immunoblotting. DENV capsid protein was detected using a guinea pig polyclonal antibody and β-actin was detected using a mouse monoclonal antibody. Data from 3 independent experiments were averaged and plotted. Bars indicate standard error of the mean.

DENV is a related flavivirus that is best known for the serious hemorrhagic disease that it causes following mosquito-borne transmission. Infection by DENV can result in vascular leakage by affecting tight junction permeability through a process involving cytokines [21]. For example, macrophage migration inhibitory factor, which is secreted by virus-infected cells, can directly affect tight junction permeability by activating MAP kinase pathways or indirectly by inducing monocytes to secrete tumor necrosis factor α (TNFα) and other cytokines that influence the barrier function of endothelial cells. DENV has also been shown to cause neuroinvasive disease which requires the virus to breach the blood brain barrier (reviewed in [22]). In contrast to WNV, DENV infection did not significantly alter the localization tight junction membrane proteins such as claudin-1 (Figure 6A) or JAM-1 (data not shown). A discernable reduction in claudin-1 protein levels (Figure 6B) was observed but this decrease was very small compared to the loss of claudin-1 in WNV-infected cells. Similarly, JAM-1 and occludin protein levels were not significantly affected by DENV infection.

### Expression of WNV capsid protein does not cause degradation of tight junction membrane proteins

**Figure 7:**
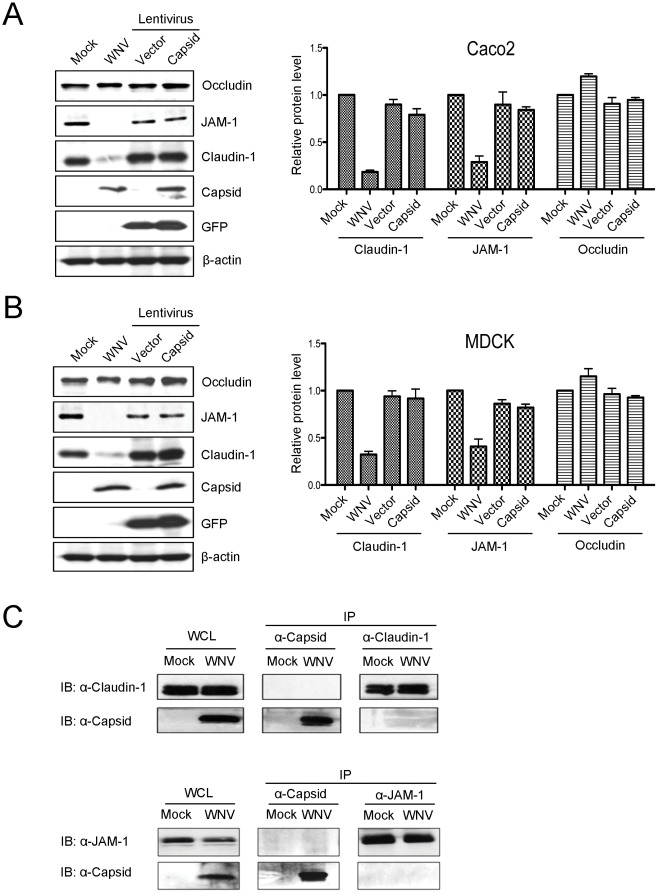
Expression of capsid in the absence of other WNV proteins does not cause degradation of tight junction proteins. CACO-2 (A) and MDCK (B) cells were infected with WNV or transduced with lentiviruses encoding GFP alone (Vector) or GFP and WNV capsid (Capsid). Forty eight hours post-infection/transduction, lysates were subjected to immunoblot analyses. Data from three independent experiments were used to determine the normalized level (to β-actin) of claudin-1, JAM-1, occludin in each sample. C. MDCK cells were infected with WNV and 48 hours later, lysates were subjected to immunoprecipitation (IP) with rabbit anti-capsid, anti-JAM-1 or mouse anti-claudin-1 antibodies followed by immunoblotting with antibodies to claudin-1, JAM-1 or capsid. WCL = whole cell lysate.

**Figure 8:**
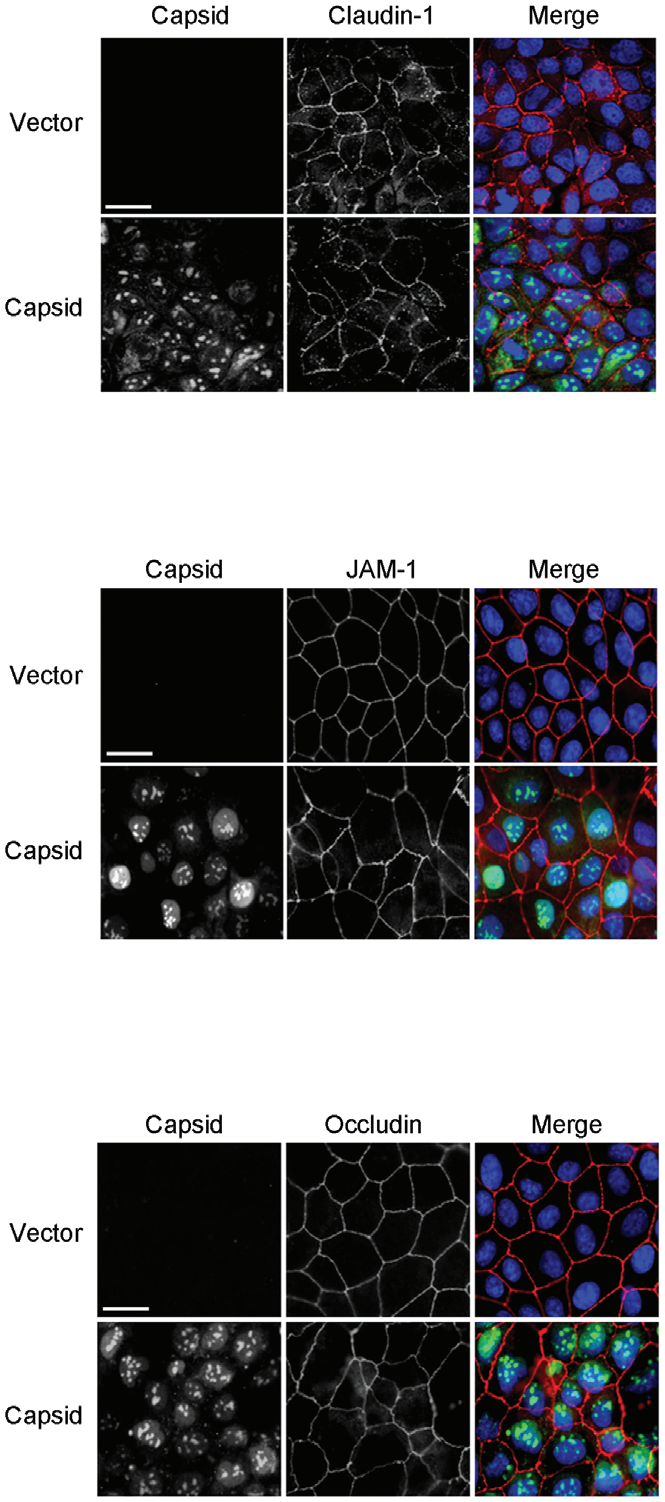
Capsid expression does not result in redistribution of claudin-1 or JAM-1. MDCK cells were transduced with vector or capsid-expressing lentiviruses and at 48 hour post transduction, were processed for indirect immunofluorescence using mouse anti-claudin-1, mouse anti-occludin, rabbit anti-JAM-1, and rabbit or guinea pig anti-WNV capsid. Primary antibodies were detected using donkey anti-mouse Alexa546, donkey anti-rabbit Alexa647, and goat anti-guinea pig Alexa546 secondary antibodies. Nuclei were counter stained with DAPI. Images were captured using a Leica TCS SP5 confocal scanning microscope. Size bar = 10 µm.

Finally, we endeavoured to understand how the WNV capsid protein which was recently reported as the virus antigen that disrupts tight junction barrier function [5], interacts with the dynamin-dependent endocytosis machinery. As the first step in this process, it was necessary to confirm that expression of capsid protein in the absence of other WNV proteins results in degradation of claudin-1. For these experiments, we used lentiviral pseudoparticles to transduce CACO-2 and MDCK cells with a cassette encoding the mature form of WNV capsid protein. In contrast to Medigeshi et al [5], we did not observe significant degradation of claudin-1 or JAM-1 in capsid-expressing cells (Figure 7A, B), nor was there any appreciable loss of tight junction membrane proteins from the cell surface (Figure 8). We also examined if capsid interacts with claudin-1 and/or JAM-1 in WNV-infected cells. Data from reciprocal co-immunoprecipitation experiments indicate that capsid does not form a stable complex with either of these proteins (Figure 7C). Moreover, consistent with what was observed in WNV-infected cells, significant colocalization between capsid and claudin-1 or JAM-1 in the transduced cells was not evident (Figure 8).

## Discussion

Tight junctions are highly dynamic structures whose organization can be altered in response to physiological and pathological situations. With respect to the latter, it is evident that the pathogenic effects of many viruses are due to loss of tight junction barrier function. Indeed, disruption of these structures is associated viral spread, circulation shock and infiltration of immune cells into compartments such as alveolar space and the central nervous system. Interestingly, there are a wide variety of mechanisms by which viruses can negatively impact tight junctions. For example, the E protein of the severe acute respiratory syndrome coronavirus interferes with lung epithelial tight junction assembly by binding to the PDZ domain of PALS1 [2]. Similarly, NS1 proteins from highly virulent strains of avian influenza virus bind and sequester PDZ domain-containing proteins such Scribble and Dlg1, MAGI-1, MAGI-2 and MAGI-3 resulting in their mislocalization from tight junctions [3]. As well as allowing influenza viruses to cross the epithelium, loss of tight junction barrier function is a major underlying factor in pulmonary edema. The examples cited above involve scenarios in which virus replication and expression of viral proteins are required for interfering with tight junctions. In contrast, the lethal circulation shock caused by Ebola virus may occur in the absence of virus replication or interaction of virus proteins with host cells. Disruption of tight junctions in this case may be due to binding of virus particles to endothelial cells which in turn activates them resulting in loss of barrier function [4].

To cause systemic infections and gain access to the central nervous system, WNV must cross epithelial and endothelial cell layers after arthropod-mediated transmission. There are numerous reports indicating that WNV infection compromises the integrity of the blood brain barrier [6], [7], [8], [23] and collectively, these data suggest that both viral and host factors are involved. With respect to the latter, WNV infection induces expression of matrix metalloproteinases such as MMP9, a host factor that is necessary for viral infection of the central nervous system [8]. One theory is that MMP9 compromises the blood brain barrier by degrading the extracellular matrix. This scenario does not rule out virus-mediated effects on tight junctions nor transcytosis as being important for crossing this barrier because once the virus breaches the endothelium, it must still traverse the extracellular matrix before it can access the central nervous system. Immunomodulatory cytokines are also thought to play a role in WNV neuroinvasion. For example, WNV infection of immune cells results in secretion of TNFα which dampens viral replication in peripheral tissues [24]. However, this anti-viral cytokine also induces endocytosis of the tight junction membrane protein occludin [25] which may inadvertently facilitate transmission of the virus across the blood brain barrier. Similarly, cytokines released from DENV-infected cells can directly or indirectly influence the permeability of endothelial tight junction complexes [21], [22].

While it is clear that flavivirus infection can negatively impact tight junctions, there is controversy as to whether or not they directly affect the expression and/or degradation of tight junction proteins. In the present study, we report for the first time, the results from a carefully controlled and coordinated study on the effects of WNV infection on tight junction membrane proteins in both epithelial and endothelial cells. Our results are in partial agreement with Medigeshi *et al* in that WNV infection results in degradation of claudin-1 protein but not occludin or ZO-1. Moreover, we observed that infection of endothelial cells (HUVECs) results in loss of claudin-1 and JAM-1 proteins. While we cannot reconcile the result of Verma *et al*. who did not observe loss of tight junction proteins in WNV-infected human brain microvascular endothelial cells, our present results and those of Medigeshi *et al*. indicate that viral infection induces loss of tight junction membrane proteins in both epithelial and endothelial cells.

Unlike Medigeshi *et al* however, we saw no evidence that expression of capsid protein alone affected levels of claudin-1 (or JAM-1); despite the fact that in both studies, capsid protein from the NY99 strain of WNV was employed. The apparent discrepancy in our results may be related to the fact that different expression systems were employed. Whereas Medigeshi *et al* used stably transfected cells expressing capsid protein for their experiments, we used lentiviral transduction to induce robust transient expression of capsid in cells which arguably, more closely parallels the expression kinetics of capsid protein in WNV-infected cells. Co-immunoprecipitation experiments failed to detect interaction between capsid and tight junction membrane proteins nor did we observe significant localization between these proteins and capsid in infected cells. Thus it seems unlikely that capsid protein is directly involved in targeting claudin-1 or JAM-1 to lysosomes.

Upon closer examination of the data from Medigeshi *et al*, we noticed that some claudin protein degradation occurred when epithelial cells were infected with subviral particles that lack the capsid gene, an observation which was attributed to the possibility that there was sufficient residual capsid protein in the virus particles to induce claudin degradation or that a redundant, capsid-independent claudin turnover mechanism is also involved [5]. These scenarios seem unlikely to us, however, it cannot be ruled out that capsid protein is required, but not sufficient for degradation of tight junction membrane proteins in WNV-infected cells.

Collectively, our data suggest that the primary mechanism for loss of claudin-1 and JAM-1 in WNV-infected cells involves endocytosis from the cell surface. Because capsid protein does not appear to be physically involved in this process, we hypothesize that WNV replication and/or assembly triggers a signalling event that leads to endocytosis of a subset of tight junction membrane proteins. Endocytosis of these proteins is in fact a normal process that is used to regulate the permeability of tight junctions in response to physiological stimuli [1]. For example, inflammatory cytokines such as TNFα induce caveolae-dependent endocytosis of occludin [25]. Even though cytokine expression and secretion is a common cellular response to virus infection, it is unlikely that this is the primary mechanism by which WNV induces lysosomal degradation of selected tight junction membrane proteins in epithelial cells at least. First, we are unaware of any data showing that WNV infection of MDCK and CACO-2 cells results in production of proinflammatory cytokines. Moreover, infection of these cells with other RNA viruses does not significantly affect production of cytokines such as TNFα and interleukins [26], [27]. Second, while treatment of CACO-2 cells with TNFα or IL-1β can lead to tight junction failure through degradation of occludin and possibly other mechanisms [28], exposure to IL-1β actually increases levels of claudin-1 [29]. In contrast, we observed that in WNV-infected epithelial cells, occludin levels remain stable while claudin-1 and JAM-1 protein levels are significantly lower.

In summary, it is likely that WNV employs multiple strategies to cross polarized cell layers. These include; stimulating endocytosis and degradation of key transmembrane proteins of the tight junction; inducing production of matrix metalloproteases that degrade basement membranes; stimulating immune cells and possibly endothelial cells to secrete proinflammatory cytokines that disrupt tight junctions. A clearer understanding how WNV causes these events may lead to therapeutic treatments that block viral spread or design of attenuated vaccine strains.

## Materials and Methods

### Reagents and antibodies

The following reagents were purchased from the respective suppliers: protein A-sepharose, protein G-sepharose from GE Healthcare Bio-Sciences AB (Piscataway, NJ); general lab chemicals, DMSO, Nocodazole, Dynasore, Paclitaxel and Latrunculin B from Sigma-Aldrich (St. Louis, MO); Complete™ EDTA-free protease inhibitor cocktail, FuGENE 6 transfection regent from Roche Diagnostics (Laval, Quebec); ProLong® Gold Antifade reagent with 4′-6-diamidino-2-phenylindole (DAPI), media and fetal bovine serum (FBS) for cell culture from Invitrogen; Pierce BCA Protein Assay Kit from Thermo Scientific (Rockford, IL); High Capacity RNA-to-cDNA Master Mix and Fast SYBR Green Master Mix from Applied Biosystems (Streetsville, ON); qScript One-Step SYBR Green qRT-PCR Kit from Quanta Biosciences (Gaithersville, MD); HEK-293T, CACO-2 and MDCK cells from the American Type Culture Collection (Manassas, VA). Human umbilical vein endothelial cells (HUVECs) isolated from individual umbilical cords as described [30] were obtained from Dr. Denise Hemmings (Obstetrics & Gynaecology, University of Alberta). These primary cells were used for experiments between passages 2–6.

The following primary antibodies were obtained from the following sources: claudin-1 specific mouse monoclonal antibodies used respectively for immunoprecipitation/immunoblotting and indirect immunofluorescence were from Invitrogen (Carlsbad, CA) and Santa Cruz Biotechnology (Santa Cruz, CA); mouse monoclonal antibodies to β-actin from Abcam (Cambridge, MA); rabbit and guinea pig polyclonal antibodies to WNV capsid protein were generated in this laboratory [10], [31]; rabbit polyclonal antibody to JAM-1 and mouse monoclonal to occludin from Invitrogen (Carlsbad, CA); mouse monoclonal antibodies against West Nile virus proteins NS3/2B from and R&D Systems (Minneapolis, MN); rabbit polyclonal antibody to green fluorescent protein (GFP) was from Dr. L. Berthiaume (University of Alberta, Edmonton, Canada). Pooled human anti-dengue virus (DENV)-2 convalescent sera [32] was kindly provided by Dr. Robert Anderson (Dalhousie University, Halifax, Canada). Polyclonal antibodies to DENV-2 capsid protein were obtained by immunization of guinea pigs (Pocono Rabbit Farm & Laboratory, Canadensis, PA) with a synthetic peptide corresponding to the 20 amino terminal amino acid residues of capsid protein coupled to keyhole limpet hemocyanin.

The following secondary antibodies were obtained from the following sources: Donkey anti-human IgG conjugated to Texas Red, goat anti-rabbit and goat anti-mouse IgG conjugated to horseradish peroxidase (Jackson ImmunoResearch Laboratories, West Grove, PA); Donkey anti-rabbit IgG conjugated to Alexa488 and Donkey anti-rabbit IgG conjugated to Alexa647, donkey anti-mouse IgG conjugated to Alexa546 or Alexa488, goat anti-guinea pig IgG conjugated to Alexa546, donkey anti-mouse conjugated to Alexa680 and donkey anti-rabbit conjugated to Alexa800 (Invitrogen, Carlsbad, CA).

### Cell culture and virus infection

CACO-2, MDCK and HEK293T cells were cultured in Dulbecco's modified Eagle's medium (DMEM) containing 10% heat-inactivated FBS, 4.5 g/liter D-glucose, 2 mM glutamine, 25 mM HEPES, 110 mg/liter sodium pyruvate, 1% penicillin-streptomycin. HUVECs were cultured in M199 containing Earle's salts, 10% heat-inactivated FBS, L-glutamine, NaHCO3, 1% penicillin-streptomycin plus 1% of endothelial cell growth supplement.

WNV strain NY99 and DENV-2 were kindly provided by Dr. Mike Drebot at the Public Health Agency of Canada (Winnipeg, MB, Canada). WNV manipulation was performed in the Glaxo CL-3 facility (University of Alberta) and DENV was handled under CL-2 conditions.

Unless otherwise indicated, cells were infected with viruses at multiplicity of infection (MOI) of 5. MDCK cells grown on coverslips or in p35 dishes were infected with WNV for 48 h and then processed for indirect immunofluorescence or co-immunoprecipitation. Where indicated, WNV-infected or mock treated MDCK or CACO-2 cell cultures were treated with nocodazole (10 µM), Dynasore (10 µM), Paclitaxel (1 µM) or Latrunculin B (10 µM). The drugs were added at 24 h post infection and the cells were incubated for 8 h before processing for immunoblot or indirect immunofluorescence analyses.

HUVEC were passaged in 0.2% gelatin-coated 25 cm^2^ flasks. For infection experiments, cells (4×10^5^) were seeded in gelatin-coated p35 dishes and allowed to attach overnight. The next day, medium was replaced with 0.5 ml of WNV stock diluted in serum-free M199 medium for 1 h after which the virus was removed. Cells were then washed twice with PBS before fresh growth medium was then added. Infected HUVECs were analyzed between 2–72 h post-infection.

### Preparation and use of lentiviruses encoding WNV capsid

A PCR-generated WNV capsid cDNA was subcloned into the *Spe*I and *Xho*I sites of the plasmid pTRIP-CMV-MCS-IRES-AcGFP; which was derived by replacing the red fluorescent protein cassette of pTRIP-CMV-IRES-tagRFP [33] with AcGFP using *Nhe*I and *Sac*II. The resulting plasmid pTRIP-IRES-AcGFP-Cap, directs independent expression of AcGFP and capsid. To produce infectious lentiviral pseudoparticles, HEK293T cells (2.5×10^6^) grown in 100 mm-diameter dishes were co-transfected with pTRIP-IRES-AcGFP-Cap (5.6 µg) or pTRIP-IRES-AcGFP (5.6 µg), pGag-Pol (5.6 µg) and pHCMV-VSVG (1.6 µg) [33] using Fugene 6 transfection reagent. Forty eight hours later, polybrene (4 µg/ml) and HEPES (20 mM) were added to harvested lentivirus-containing cell culture supernatants which were then passed through 0.45 µm filter, aliquotted and then stored at −80°C or used to transduced CACO-2 or MDCK cells in 6 well dishes. Typically, lentiviral stocks were diluted 1∶10 in DMEM containing 3% FBS, polybrene (4 µg/ml polybrene) and HEPES (20 mM). Cells were then spinoculated by centrifugation at 1200 rpm in an Eppendorf A-4-62 rotor for 1 h at 37°C after which the plates were transferred to a 37°C incubator. After 6 h, the media were replaced with DMEM containing 10% FBS. Unless otherwise indicated, transduced cells were analyzed 48 h post-transduction.

### Immunoblotting

Virus-infected or lentivirus-transduced cells were washed twice with cold PBS on ice, then lysed in RIPA buffer (50 mM Tris-HCl [pH 7.4], 150 mM NaCl, 1% Triton x-100, 1% sodium deoxycholate, 0.1% sodium dodecylsulfate [SDS], 1 mM EDTA) containing a cocktail of protease inhibitors. Cell lysates were incubated on ice for 30 min and then centrifuged at 12,000×g for 15 min at 4°C after which protein concentrations in the supernatants were quantified by BCA assay. Equivalent amounts of proteins (20 µg/sample) were resolved by SDS-PAGE, transferred to immobilon-polyvinylidene fluoride (PVDF) membranes and then detected by immunoblotting. Quantification of the proteins detected in the immunoblots using fluorescently tagged secondary antibodies was performed by using a Licor Odyssey Infrared Imaging System (Lincoln, NE) using the protocol posted at http://biosupport.licor.com. Relative levels of claudin-1, JAM-1 and occludin (normalized to β-actin) were determined using Odyssey Infrared Imaging System 1.2 Version software.

### Indirect immunofluorescence

MDCK and CACO-2 cells grown on coverslips were processed for indirect immunofluorescence microscopy 48 h after infection with WNV or transduction with the lentiviral vector. Cells were washed twice in PBS containing 0.5 mM Ca^2+^ and 1.0 mM Mg^2+^ (PBSCM) and then fixed in 4% paraformaldehyde for 20 min. Samples were quenched with PBS containing 50 mM ammonium chloride and then washed twice with PBSCM. Cell membranes were permeabilized with PBS containing 0.2% Triton X-100 for 5 min and then nonspecific antibody binding sites were blocked by incubating with PBSCM solution containing 1% BSA for 30 min at room temperature (RT). For samples that were stained with rabbit anti-JAM-1, cells were fixed with ethanol at 4°C for 30 minutes followed by treatment with cold acetone for 5 minutes at RT. Fixed cells were washed three times, followed by incubation with pooled human anti-DENV sera (1∶2500), mouse anti-claudin-1 (1∶300), (Santa Cruz), mouse anti-occludin (1∶300), rabbit anti-JAM-1 (1∶300), mouse anti-NS3/2B (1∶500) and/or rabbit anti-WNV capsid (1∶300) for 2 h at RT. Primary antibodies were detected with Alexa Fluor 546-conjugated Donkey anti-mouse, Alexa Fluor 647-conjugated donkey anti-rabbit, Alexa Fluor 488-conjugated donkey anti-rabbit secondary antibodies for 1 h at RT. Coverslips were mounted onto microscope slides using ProLong® Gold Antifade containing DAPI and samples were examined using a Leica TCS SP5 confocal microscope. Captured images were processed using Image J and LAS AF Lite softwares.

### Co-immunoprecipitation

MDCK cells (3×10^5^) seeded into P35 dishes, were infected the next day with WNV. After 48 hours, the cells were washed twice with cold PBS and then lysed with NP-40 lysis buffer (150 mM NaCl, 2 mM EDTA, 1% Nonidet P-40, 50 mM Tris-HCl [pH 7.2], 1 mM fresh dithiothreitol) containing protease inhibitors on ice for 30 min. Lysates were clarified by centrifugation for 15 min at 12,000×g in a microcentrifuge at 4°C. Small aliquots of the clarified lysates were kept for loading controls. The remaining lysates were pre-cleared with protein G-Sepharose or protein A-Sepharose beads for 1 h at 4°C before sequential incubation with mouse anti-claudin-1 or rabbit anti-capsid or JAM-1 antibodies for 3 h and then protein G-Sepharose beads or protein A-Sepharose beads for 2 h at 4°C. Immunoprecipitates were washed three times with lysis buffer before the bound proteins were eluted by boiling in protein sample buffer. Proteins were separated by SDS-PAGE and transferred to PVDF membranes for immunoblotting.

### Quantitative PCR analysis

Expression of junction protein genes in WNV-infected CACO-2 cells was measured using quantitative RT-PCR of total RNA. Data was obtained using RNA extracted from two batches of independently infected cells. Total RNA from WNV-infected cells harvested at 24 h, 48 h and 72 h post-infection was isolated with TRI Reagent® (Ambion). RNA samples were adjusted to 100 ng/µl by dilution in sterile RNAse/DNAse free water. Relative levels of claudin-1, claudin-3, claudin-4, JAM1, ZO-1 and occludin mRNAs as well as WNV genomic RNA were determined using primers listed in Table 1. All genes of interest were analysed in a two-step as well as in a one-step reverse transcriptase (RT-) PCR approach.

**Table 1:** List of Oligonucleotide Primers.

Primer	Sequence	T_m_ (°C)*	Reference
Claudin-1 Forward	CCAACGCGGGGCTGCAGCT	55	[35]
Claudin-1 Reverse	TTGTTTTTCGGGGACAGGA	44	
Claudin-3 Forward	CTGCTCTGCTGCTCGTGTCC	53	[36]
Claudin-3 Reverse	TTAGACGTAGTCCTTGCGGTCGTAG	54	
Claudin-4 Forward	GGCTGCTTTGCTGCAACTGTC	51	[36]
Claudin-4 Reverse	GAGCCGTGGCACCTT ACACG	53	
Occludin Forward	TCAAACCGAATCATTATGCACCA	47	[36]
Occludin Reverse	AGATGGCAATGCACATCACAA	45	
JAM1 Forward	ACCAAGGAGACACCACCAGAC	51	
JAM1 Reverse	GAGGCACAAGCACGATGAGC	51	
ZO-1 Forward	CAAGATAGTTTGGCAGCAAGAGATG	51	[37]
ZO-1 Reverse	ATCAGGGACATTCAATAGCGTAGC	51	
GAPDH Forward	GAAATCCCATCACCATCTTCCAGG	52	
GAPDH Reverse	GAGCCCCAGCCTTCTCCATG	53	
WNV Forward	TCTGCGGAGAGTGCAGTCTGCGAT	56	[38]
WNV Reverse	TCAGCGATCTCTCTC CACCAAAG	52	

* adjusted for 50 mM salt concentration.

In the two-step reaction setup, cDNA was generated from 500 ng of RNA (5 µl of diluted total RNA) using High Capacity RNA-to-cDNA Master Mix (Applied Biosystems, Streetsville, ON) with random primers in a reaction volume of 20 µl. Reaction conditions were 5 min at 25°C, 30 min at 42°C and 5 min at 85°C. The resulting cDNA was diluted 1/10 in RNase free water, of which 5 µl was used for subsequent DNA amplification. The amplification was conducted with Fast SYBR Green Master Mix (Applied Biosystems, Streetsville, ON) in a reaction volume of 25 µl. Primer concentration was 10 pmol. Reaction conditions used were: 95°C for 30 sec, followed by 40 cycles of 95°C for 5 sec and 55–60°C for 30 sec (temperature for annealing/extension step dependent on primer T_m_ (Table 1)).

One-step reactions were performed in 25 µl volumes using qScript One-Step SYBR Green qRT-PCR Kit (Quanta Biosciences, Gaithersville, MD) starting with 100 ng of total RNA. Primer concentration was 10 pmol. Reaction conditions were: 50°C for 10 min, 95°C for 5 min, followed by 40 cycles of 95°C for 10 sec, 55–60°C for 20 sec and 72°C for 30 sec.

Formation of primer dimers and other unspecific products was monitored by melt curve analysis (from 55°C to 95°C). Relative quantification of gene expression and successive calculation of fold-increase of gene expression of tight junction mRNAs were normalized to GAPDH mRNA levels using the comparative cT (ΔΔcT) method [34] All experiments were conducted in triplicates, resulting in a minimum of eight data points for each gene of interest. All gene expression studies were conducted on a CFX96 (Bio-Rad, Hercules, CA) or an Mx3005P (Stratagene, LaJolla, CA) thermocycler. Statistical analysis on the data was conducted with SPSS Statistics 17.0 software (SPSS Inc. Chicago, IL). Significant variance of mRNA expression at 24, 48 and 72 h post infection was evaluated using Tamhane's T2 multiple comparison.

## References

[1] Shen L, Weber CR, Raleigh DR, Yu D, Turner JR (2011). Tight junction pore and leak pathways: a dynamic duo.. Annu Rev Physiol.

[2] Teoh KT, Siu YL, Chan WL, Schluter MA, Liu CJ (2010). The SARS coronavirus E protein interacts with PALS1 and alters tight junction formation and epithelial morphogenesis.. Mol Biol Cell.

[3] Golebiewski L, Liu H, Javier RT, Rice AP (2011). The avian influenza virus NS1 ESEV PDZ binding motif associates with Dlg1 and Scribble to disrupt cellular tight junctions.. J Virol.

[4] Wahl-Jensen VM, Afanasieva TA, Seebach J, Stroher U, Feldmann H (2005). Effects of Ebola virus glycoproteins on endothelial cell activation and barrier function.. J Virol.

[5] Medigeshi GR, Hirsch AJ, Brien JD, Uhrlaub JL, Mason PW (2009). West nile virus capsid degradation of claudin proteins disrupts epithelial barrier function.. J Virol.

[6] Verma S, Kumar M, Gurjav U, Lum S, Nerurkar VR (2010). Reversal of West Nile virus-induced blood-brain barrier disruption and tight junction proteins degradation by matrix metalloproteinases inhibitor.. Virology.

[7] Verma S, Lo Y, Chapagain M, Lum S, Kumar M (2009). West Nile virus infection modulates human brain microvascular endothelial cells tight junction proteins and cell adhesion molecules: Transmigration across the in vitro blood-brain barrier.. Virology.

[8] Wang P, Dai J, Bai F, Kong KF, Wong SJ (2008). Matrix metalloproteinase 9 facilitates West Nile virus entry into the brain.. J Virol.

[9] Xu Z, Anderson R, Hobman TC (2011). The capsid-binding nucleolar helicase DDX56 is important for infectivity of West Nile virus.. J Virol.

[10] Hunt TA, Urbanowski MD, Kakani K, Law LM, Brinton MA (2007). Interactions between the West Nile virus capsid protein and the host cell-encoded phosphatase inhibitor, I2PP2A.. Cell Microbiol.

[11] Takahashi S, Iwamoto N, Sasaki H, Ohashi M, Oda Y (2009). The E3 ubiquitin ligase LNX1p80 promotes the removal of claudins from tight junctions in MDCK cells.. J Cell Sci.

[12] Ivanov AI, Nusrat A, Parkos CA (2004). Endocytosis of epithelial apical junctional proteins by a clathrin-mediated pathway into a unique storage compartment.. Mol Biol Cell.

[13] Daugherty BL, Mateescu M, Patel AS, Wade K, Kimura S (2004). Developmental regulation of claudin localization by fetal alveolar epithelial cells.. Am J Physiol Lung Cell Mol Physiol.

[14] Hinshaw JE, Schmid SL (1995). Dynamin self-assembles into rings suggesting a mechanism for coated vesicle budding.. Nature.

[15] Macia E, Ehrlich M, Massol R, Boucrot E, Brunner C (2006). Dynasore, a cell-permeable inhibitor of dynamin.. Dev Cell.

[16] Caviston JP, Holzbaur EL (2006). Microtubule motors at the intersection of trafficking and transport.. Trends Cell Biol.

[17] Jin M, Snider MD (1993). Role of microtubules in transferrin receptor transport from the cell surface to endosomes and the Golgi complex.. J Biol Chem.

[18] Roeth JF, Williams M, Kasper MR, Filzen TM, Collins KL (2004). HIV-1 Nef disrupts MHC-I trafficking by recruiting AP-1 to the MHC-I cytoplasmic tail.. J Cell Biol.

[19] Scheel J, Matteoni R, Ludwig T, Hoflack B, Kreis TE (1990). Microtubule depolymerization inhibits transport of cathepsin D from the Golgi apparatus to lysosomes.. J Cell Sci.

[20] Jones SM, Howell KE, Henley JR, Cao H, McNiven MA (1998). Role of dynamin in the formation of transport vesicles from the trans-Golgi network.. Science.

[21] Chuang YC, Lei HY, Liu HS, Lin YS, Fu TF (2011). Macrophage migration inhibitory factor induced by dengue virus infection increases vascular permeability.. Cytokine.

[22] Sips GJ, Wilschut J, Smit JM (2011). Neuroinvasive flavivirus infections.. Rev Med Virol.

[23] Wang T, Town T, Alexopoulou L, Anderson JF, Fikrig E (2004). Toll-like receptor 3 mediates West Nile virus entry into the brain causing lethal encephalitis.. Nat Med.

[24] Diamond MS, Klein RS (2004). West Nile virus: crossing the blood-brain barrier.. Nat Med.

[25] Marchiando AM, Shen L, Graham WV, Weber CR, Schwarz BT (2010). Caveolin-1-dependent occludin endocytosis is required for TNF-induced tight junction regulation in vivo.. J Cell Biol.

[26] Cuadras MA, Feigelstock DA, An S, Greenberg HB (2002). Gene expression pattern in Caco-2 cells following rotavirus infection.. J Virol.

[27] Grone A, Fonfara S, Baumgartner W (2002). Cell type-dependent cytokine expression after canine distemper virus infection.. Viral Immunol.

[28] Al-Sadi R, Boivin M, Ma T (2009). Mechanism of cytokine modulation of epithelial tight junction barrier.. Front Biosci.

[29] Al-Sadi RM, Ma TY (2007). IL-1beta causes an increase in intestinal epithelial tight junction permeability.. J Immunol.

[30] Zimmerman GA, McIntyre TM, Prescott SM (1985). Thrombin stimulates the adherence of neutrophils to human endothelial cells in vitro.. J Clin Invest.

[31] Beatch MD, Hobman TC (2000). Rubella virus capsid associates with host cell protein p32 and localizes to mitochondria.. J Virol.

[32] He RT, Innis BL, Nisalak A, Usawattanakul W, Wang S (1995). Antibodies that block virus attachment to Vero cells are a major component of the human neutralizing antibody response against dengue virus type 2.. J Med Virol.

[33] Schoggins JW, Wilson SJ, Panis M, Murphy MY, Jones CT (2011). A diverse range of gene products are effectors of the type I interferon antiviral response.. Nature.

[34] Livak KJ, Schmittgen TD (2001). Analysis of relative gene expression data using real-time quantitative PCR and the 2(-Delta Delta C(T)) Method.. Methods.

[35] Chang TL, Ito K, Ko TK, Liu Q, Salto-Tellez M (2010). Claudin-1 has tumor suppressive activity and is a direct target of RUNX3 in gastric epithelial cells.. Gastroenterology.

[36] Michikawa H, Fujita-Yoshigaki J, Sugiya H (2008). Enhancement of barrier function by overexpression of claudin-4 in tight junctions of submandibular gland cells.. Cell Tissue Res.

[37] Drago S, El Asmar R, Di Pierro M, Grazia Clemente M, Tripathi A (2006). Gliadin, zonulin and gut permeability: Effects on celiac and non-celiac intestinal mucosa and intestinal cell lines.. Scand J Gastroenterol.

[38] Lanciotti RS, Kerst AJ, Nasci RS, Godsey MS, Mitchell CJ (2000). Rapid detection of west nile virus from human clinical specimens, field-collected mosquitoes, and avian samples by a TaqMan reverse transcriptase-PCR assay.. J Clin Microbiol.

